# Quantitative evaluation method for clarifying ankle plantar flexion angles using anterior drawer and inversion stress tests: a cross-sectional study

**DOI:** 10.1186/s13047-019-0337-y

**Published:** 2019-05-03

**Authors:** Takanori Kikumoto, Kazuma Akatsuka, Emi Nakamura, Wataru Ito, Ryo Hirabayashi, Mutsuaki Edama

**Affiliations:** 1 Institute for Human Movement and Medical Sciences, Physical Therapy Department, Niigata University of Health and Welfare, 1398 Shimami-cho, Kita-ku, Niigata-shi, Niigata, 950-3198 Japan; 2 Johto Orthopaedic Clinic, 6-7-6 Higashidori, Akita-shi, Akita, 010-0003 Japan

**Keywords:** Anterior talofibular ligament, Chronic ankle instability, Telos stress device, Diagnostic ultrasound imaging systems

## Abstract

**Background:**

Chronic ankle instability (CAI) may result from repeated, frequent ankle sprains during sports activities. Manual examination for CAI is conducted; however, quantitative methods for the evaluation of CAI have not been established, and the reproducibility of the amount of stress is low. This cross-sectional study aimed to use a stress device and ultrasound for the quantitative evaluation of the change in the length of the anterior talofibular ligament (ATFL) during simulated anterior drawer and ankle inversion stress tests.

**Methods:**

Questionnaires were provided to 160 healthy college students (86 men, 74 women; 320 ankles). We extracted two groups from them: control subjects without a history of ankle injury (*n* = 64 ankles) and subjects with CAI (*n* = 54 ankles). We calculated the change in the length of the ATFL with anterior drawer and inversion stress tests at ankle joint plantar flexions of 0°, 20°, and 45° using ultrasound images.

**Results:**

The anterior length change rates were significantly higher in the CAI group than in the control group at ankle joint plantar flexions of 20° and 45° in men (*P* < 0.05). The inversion length change rates were significantly higher in the CAI group at ankle joint plantar flexion of 20° in men (*P* < 0.05). No significant between-group difference in the anterior and inversion length change rates was observed in women.

**Conclusions:**

Stress ultrasound revealed greater length changes in the ATFL in the CAI group than in the control group. The stress test may be useful at ankle joint plantar flexion of 20° for men.

## Background

Ankle sprains are among the most frequently occurring sports injuries, accounting for approximately 12–20% of all injuries [1–3]. Ankle sprains often occur in cutting and jumping situations during sports activities [4, 5] and are roughly categorized into two types: internal and external sprains. Internal sprains occur in approximately 70–77% of cases [6], whereas external sprains occur in approximately 5–14% [6, 7]. The most common causes of ankle sprains are ankle plantar flexion injuries and valgus injuries, calcaneofibular ligament (CFL) damage occurring in approximately 2% of cases [6], in contrast with anterior talofibular ligament (ATFL) damage occurring in approximately 65–73% of cases [6, 8]. The ATFL begins on the inner leading edge of the fibular lateral malleolus and attaches to the neck of the talus. The ATFL limits plantar flexion and ankle inversion [8] and may be easily and more frequently damaged with inversion ankle sprains. The fibular lateral malleolus is located more distally than the medial malleolus. Additionally, the lateral ligament of the ankle joint is fragile compared with the medial ligament [9], which may explain why the ATFL has higher rates of injury with inversion ankle sprains. The talocrural joint is most anatomically stable in the dorsiflexion position; however, bone stability is reduced in the plantar flexion position. ATFL stability greatly contributes to the overall stability of the ankle joint [9].

In addition to the high incidence of inversion ankle sprains, the rate of recurrent trauma and injury to the ankle is approximately 73.5% [10]. In many cases, inversion ankle sprains tend to be considered minor injuries [11]. However, when rehabilitation is insufficient and ankle sprains are recurrent, sequelae such as chronic ankle instability (CAI) are likely to occur. As ankle joint instability is an indicator of CAI [12], post-injury rehabilitation is crucial. Without rehabilitation, CAI can become a great hindrance to participation in sports activities. Hertel reported that CAI occurs because of repetitive ankle sprains caused by a combination of structural and functional instabilities [13, 14]. According to the International Ankle Consortium, the criteria for CAI include the following: a history of one or more ankle sprains; an ankle with a history of “giving way,” which indicates joint instability; and an ankle condition that meets the criteria in recommended questionnaires such as the Cumberland Ankle Instability Tool and Identification of Functional Ankle Instability questionnaire. The criteria for excluding a diagnosis of CAI are a history of no more than one (initial) joint sprain within a 1-year period, a history of ankle sprain within 3 months, and a history of fracture or surgery on the lower limbs. Functional instability of the ankle joint is diagnosed using scoring instruments or subjective feelings of ankle instability as well as other subjective symptoms in daily life. Sports surgery performed as treatment for functional instability can also be used to diagnose functional instability of the ankle [13]. In many cases, evaluations of ankle instability are objectively performed using manual examination, radiography, or magnetic resonance imaging (MRI).

In most objective manual examinations, it is impossible to create a constant load during manual operation tests; however, the two manual ankle examination methods that are currently used to identify sprains are the anterior drawer and inversion stress tests. Stress X-ray photography using the Telos stress device (Telos) can make the load constant but exposes the patient to radiation, which is a disadvantage of the test. MRI is used to visualize muscle and ligamentous tissues but is limited by its high cost and low versatility because of the massive size of the equipment. In recent years, ultrasound imaging devices for these purposes have increased in popularity. These devices have begun to replace MRI because they allow changes in body tissues to be visualized in real time as manual stresses to the joints can be applied on the sports fields, in stadiums, and in outpatient clinics [15, 16]. Ultrasound is comparatively low-cost, imposes fewer restrictions on usage locations, and is very safe for patients. Lee et al. reported the usefulness of quantitative evaluations using ultrasound in combination with the anterior drawer stress test [17]. In a previous study, differences in length change rates of the fibular lateral malleolus and talus were identified among patients with a history of ankle sprains after evaluations that included anterior drawer stress test or ultrasound imaging [18, 19]. However, manual ankle joint stress tests cannot define ankle joint plantar flexion angles and are not reproducible because it is impossible to make the loads constant.

In this study, we compared a control group (subjects with no history of ankle sprain and lower limb surgery) with a CAI group (subjects with ankle joint instability) to clarify differences in length change rates of the fibular lateral malleolus and talus based on anterior drawer and inversion stress tests performed at different ankle joint plantar flexion angles using Telos and diagnostic ultrasound imaging systems.

## Methods

### Subjects

Questionnaires were provided to 160 healthy college students (86 men, 74 women; 320 ankles). The study data extraction method included the following: history of one or more ankle sprains, which are criteria for CAI according to the International Ankle Consortium; two or more episodes of an ankle “giving way”; and two or more ipsilateral ankle sprains experienced within the prior 6 months. These criteria met the standards of the Cumberland Ankle Instability Tool, which is a recommended questionnaire used for subjects who do not meet any of the criteria. CAI is identified by a score of 24 points or less out of 30 using the Cumberland Ankle Instability Tool and a score of over 11 points using the Identification of Functional Ankle Instability questionnaire. We extracted two groups from them: 54 ankles (28 men, 26 women) with CAI and 64 uninjured ankles (30 men, 34 women) participated in this study (Table 1).

**Table 1:** Characteristics of the Chronic Ankle Instability and Control Groups

	Mean ± SD			Mean ± SD		
Variable	Men		*P* Value	Women		*P* Value
	CAI (*n* = 28)	Control (*n* = 30)		CAI (*n* = 26)	control (*n* = 34)	
Age, y	20.9 ± 1.4	20.4 ± 0.8	0.098	20.4 ± 1.6	20.7 ± 2.2	0.153
Height, cm	174.2 ± 9.8	173.1 ± 9.3	0.800	158.2 ± 6.8	154.6 ± 8.8	0.753
Body weight, kg	71.0 ± 7.7	68.1 ± 9.9	0.650	58.0 ± 8.2	55.3 ± 9.5	0.538
No. of previous ankle sprains	3.1 ± 1.53	NA	NA	2.6 ± 2.38	NA	NA
Time since last sprain, mo	9.0 ± 2.90	NA	NA	10.3 ± 3.67	NA	NA

Prior to their participation, all subjects were verbally informed about the purpose and contents of this research, and written informed consent was obtained from each subject. The study was approved by the ethics committee of Niigata University of Health and Welfare (no. 17798–170,285), and the research was conducted in accordance with the tenets of the Declaration of Helsinki.

### Equipment used

In this study, we used a diagnostic ultrasound imaging system (Aplio 500; Canon Medical Systems, Tokyo, Japan) and a high-frequency linear probe (PLT-1005 BT, 10 MHz; Canon Medical Systems, Tokyo, Japan) to photograph the fibular lateral malleolus and talus. Quantitative stresses were applied to the ankle joints to simulate anterior drawer and inversion stresses using the Telos stress device (Aimedic MMT Co., Ltd., Tokyo, Japan). In addition, a joint angle meter (Takase Medical Co., Ltd., Tokyo, Japan) was used to measure the ankle joint flexion angle. All ankle angles were determined by one examiner.

### Measurement method

#### Measurements of the fibular lateral malleolus and talus at rest

**Fig. 1:**
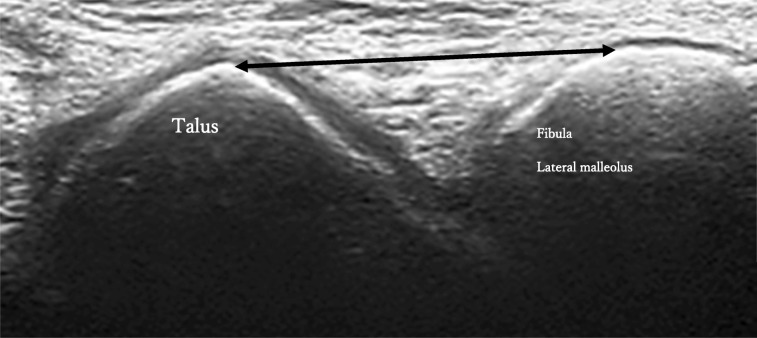
Ultrasound images of the fibular lateral malleolus and talus The probe is applied to the long axis of the fibular lateral malleolus and the anterior lateral portion of the talus as markers. We checked the anterior and posterior talus bone projections, which are drawn at sharp angles on the image. Subsequently, we checked the rounded fibular lateral malleolus and photographed them in B mode

Using diagnostic ultrasound according to the method of Singh [20], a linear probe was used to measure the distance between the fibular lateral malleolus and talus, which is the most prominent part of the proximal surface of the fibula in the anterior half of the outer anterior slope of the ankle joint (Fig. 1).

#### Anterior drawer and inversion stress test measurements

**Fig. 2:**
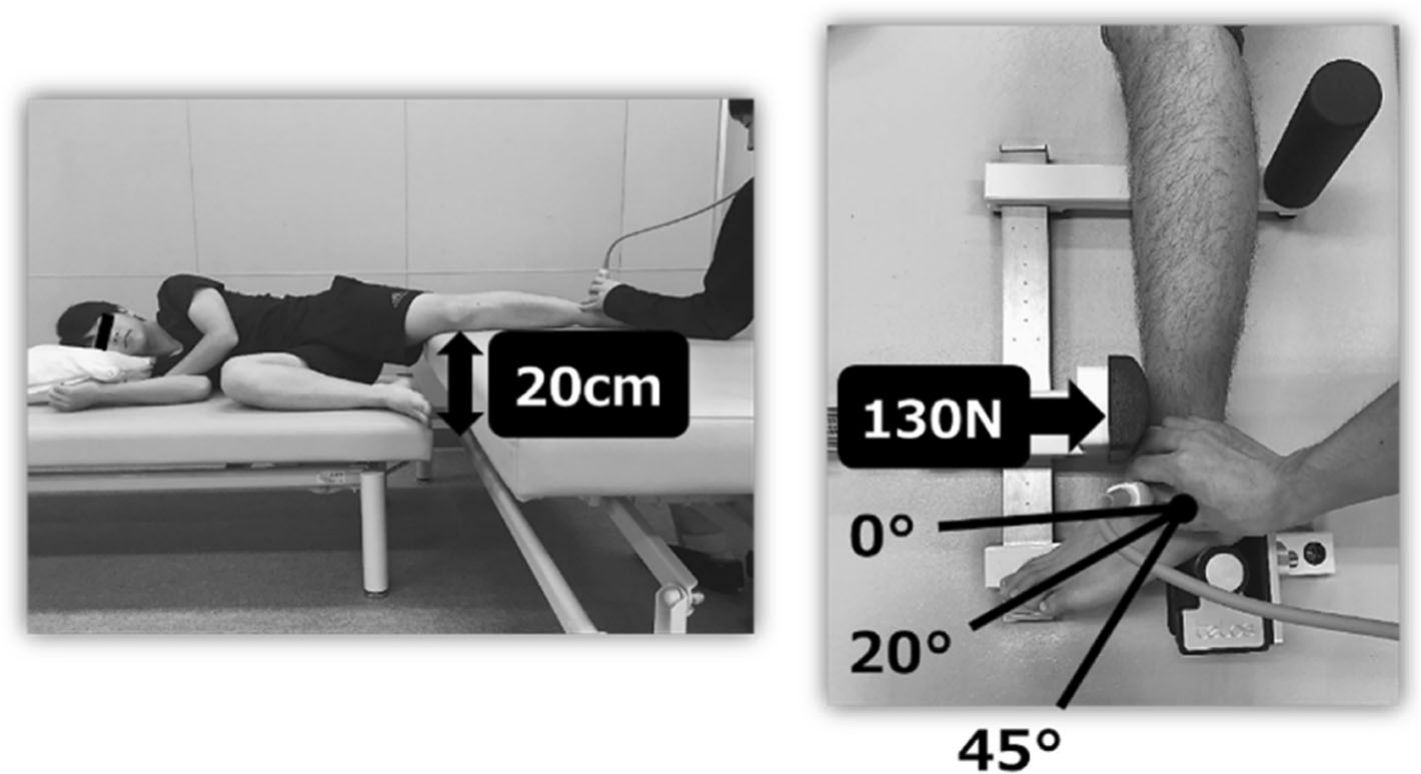
Anterior drawer stress test position and limb position To perform the anterior drawer stress test, the subject is placed on a bed in lateral position with the feet on the Telos. The posture at the time of measurement was as follows: 10° of hip joint flexion and 20° of knee joint flexion. The position of stress at ankle joint plantar flexions of 0°, 20°, and 45° was set at 5 cm proximal to the fibular lateral malleolus. The anterior drawer stress was measured under a load of 130 N or more and photographed

To perform the anterior drawer stress test, the subject was placed on a bed in lateral position with the feet on the Telos. The posture at the time of measurement was as follows: 10° of hip joint flexion and 20° of knee joint flexion. The position of stress at ankle joint plantar flexions of 0°, 20°, and 45° was set at 5 cm proximal to the fibular lateral malleolus. The anterior drawer stress test was performed with a load of 130 N or higher, and the process was photographed (Fig. 2).

**Fig. 3:**
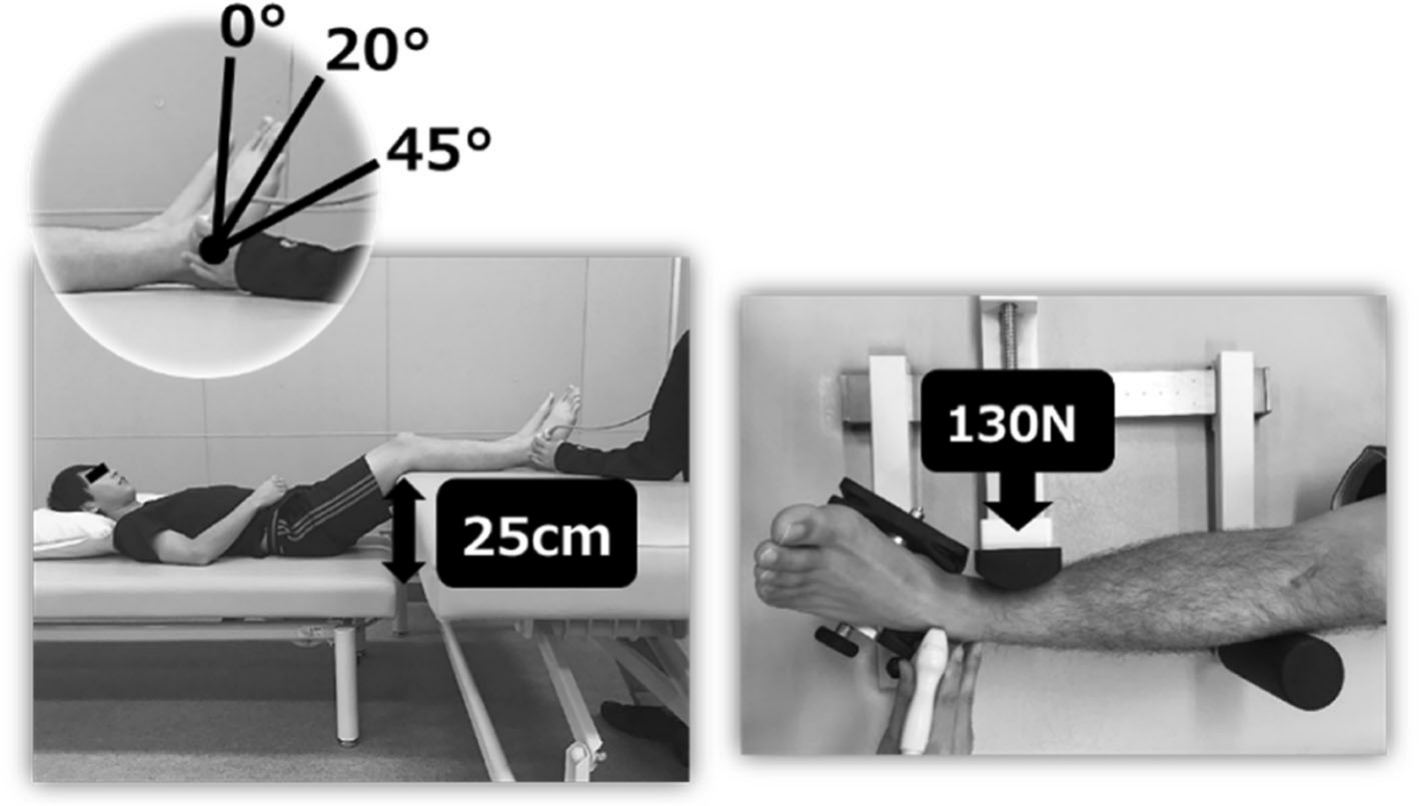
Inversion stress test position and limb position To perform the inversion stress test, the subject was placed on a bed in supine position with the feet on the Telos and the knees bent. The position of stress at ankle joint plantar flexions of 0°, 20°, and 45° was set at 5 cm proximal to the fibular lateral malleolus. The inversion stress was measured under a load of 130 N or more and photographed

To perform the inversion stress test, the subject was placed on the bed in supine position with the feet on the Telos and the knee joint at 20° of flexion. The position of stress at ankle joint plantar flexions of 0°, 20°, and 45° was set at 5 cm proximal to the fibular lateral malleolus. The inversion stress test was performed with a load of 130 N or higher, and the process was photographed (Fig. 3).

Ankle joint flexion angles were measured by three examiners using goniometers during anterior drawer and inversion stress test measurements. Based on a previous study that reported that the length change rate reached a maximum under a load of 130 N or higher, we set our load at 130 N [18].

#### Analysis method

Three ultrasound images were obtained at rest and during stress, and the recorded images were analysed using ImageJ analysis software (National Institutes of Health, Atlanta, GA, USA, 2012). Three measurements were obtained from each image, and the average value was calculated. Considering individual differences, the distances between the fibular lateral malleolus and talus at rest and during stress were measured and recorded as length change rates of the fibular lateral malleolus and talus.

$$ \mathrm{Length}\ \mathrm{Change}\ \mathrm{Rates}=\left(\mathrm{Stress}\ \mathrm{Value}-\mathrm{Resting}\ \mathrm{Value}/\mathrm{Resting}\ \mathrm{Value}\right)\times 100 $$

#### Test reproducibility

A total of 20 men (age, 20.8 ± 1.3 years; height, 175.1 ± 3.9 cm; weight, 72.3 ± 6.9 kg) and 20 women (age, 20.4 ± 1.5 years; height, 163.1 ± 3.9 cm; weight, 58.1 ± 5.6 kg) who did not have ankle joint pain were investigated with respect to measurement reproducibility of length change rates at ankle joint plantar flexion angles of 0°, 20°, and 45°. Three measurements were obtained, and the average value was used, similar to the method employed to measure length change rates of the fibular lateral malleolus and talus. Measurements were repeated at 2 days after the first measurement to verify their reproducibility.

### Statistical analyses

The sample size was performed using Ene 3.0. The required sample was determined taking as a reference the data reported by de Noronha M [21]. The distance and length change rates of the fibular lateral malleolus and talus were tested for normality using the Shapiro-Wilk test with respect to plantar flexion angles in each group. Student’s t-test and Welch’s t-test were performed after checking for normality. One-way analysis of variance without repetition was performed for each plantar flexion angle, and a posteriori tests were performed using the Tukey–Kramer method. The average of three measurements was calculated using intraclass correlation coefficients (ICC) (1, 3) to determine the reproducibility of length change rates of the fibular lateral malleolus and talus at plantar flexion angles. All statistical analyses were performed using SPSS for Windows version 20 (IBM Corp., Armonk, NY, USA), with statistical significance set at *P* < 0.05.

## Results

### Verification of measurement reproducibility

The ICCs (1, 3) for the length change rates of the fibular lateral malleolus and talus ranged from 0.875 to 0.949 (Table 2). According to previous research criteria, our measurement reproducibility was high because reproducibility is considered almost perfect when the ICC is 0.81 or higher [22].

**Table 2:** Inter-session reliability of the distance between the fibular malleolus and talus^a^

Sex	N	Ankle joint angle	ICC	Reliability
Men		0°	0.949	Almost perfect
	20	20°	0.935	Almost perfect
		45°	0.899	Almost perfect
Women		0°	0.922	Almost perfect
	20	20°	0.827	Almost perfect
		45°	0.875	Almost perfect

*ICC* intraclass correlation coefficient

^a^According to previous research criteria, the measurement reproducibility in this study was considered high because reproducibility is thought to be almost perfect when the ICC is 0.81 or more [22]

### Distance between the fibular lateral malleolus and talus at rest

#### No change in ankle joint flexion angle at rest

The average distance between the fibular lateral malleolus and talus at rest was 20.8 ± 2.6 mm in the control group and 22.6 ± 3.4 mm in the CAI group (Table 3). The values were significantly higher in the CAI group than in the control group for both men (*P* = 0.045) and women (*P* = 0.042). In a same-sex comparison, both men and women showed significantly higher values in the CAI group than in the control group. Furthermore, when compared according to sex, men in both the control and CAI groups showed significantly higher values than women in both groups.

**Table 3:** Average distance between the fibular malleolus and talus at rest

	Control	CAI	*P* value	Effect size
Men	21.73 ± 2.67 (mm)	23.51 ± 3.65 (mm)	*P = 0.045	0.06
Women	19.90 ± 2.54 (mm)	21.61 ± 3.05 (mm)	*P = 0.042	0.14
*P* value	*P = 0.046	*P = 0.040		
Effect size	0.13	0.09		

*CAI* chronic ankle instability

*Significant difference between groups (*P* < 0.05)

#### Change in ankle joint flexion angle at rest

When changing the ankle joint flexion angle at rest, the distance between the fibular lateral malleolus and talus was significantly higher in men than in women at 20° (*P* = 0.036) and 45° (*P* = 0.043) in the control group and at 45° (*P* = 0.034) in the CAI group (Table 4). The men and women in the CAI group showed significantly higher distances than those in the control group at ankle joint plantar flexions of 45° (P = 0.043) and 0° (*P* = 0.038), respectively.

**Table 4:** Average distance between the fibular malleolus and talus according to ankle joint angle at rest

Ankle joint angle	Men control (mm)	Men CAI (mm)	P value	Effect size
0°	19.62 ± 3.85	21.73 ± 4.51	*P* = 0.102	0.18
20°	22.26 ± 2.68	23.65 ± 3.98	*P* = 0.966	0.36
45°	23.14 ± 2.49	25.03 ± 3.57	*P = 0.043	0.09
Ankle joint angle	Women control (mm)	Women CAI (mm)	P value	Effect size
0°	18.32 ± 2.29	20.57 ± 4.22	*P = 0.038	0.06
20°	20.26 ± 2.17	21.80 ± 3.95	*P* = 0.832	0.22
45°	21.12 ± 2.55	22.50 ± 3.82	*P* = 0.864	0.22
0°	19.62 ± 3.85	18.32 ± 2.29	*P* = 0.917	0.29
20°	22.26 ± 2.68	20.26 ± 2.17	*P = 0.036	0.14
45°	23.14 ± 2.49	21.12 ± 2.55	*P = 0.043	0.11
Ankle joint angle	Men CAI (mm)	Women CAI (mm)	P value	Effect size
0°	21.73 ± 4.51	20.57 ± 4.22	*P* = 0.838	0.19
20°	23.65 ± 3.98	21.80 ± 3.95	*P* = 0.732	0.09
45°	25.03 ± 3.57	22.50 ± 3.82	*P = 0.034	0.06

*CAI* chronic ankle instability

*Significant difference between groups (P < 0.05)

### Length change rates of the fibular lateral malleolus and talus during the anterior drawer stress test

#### Data according to sex

Among men, length change rates between the fibular lateral malleolus and talus during the anterior drawer stress test were significantly higher in the CAI group at ankle joint plantar flexions of 20° (*P* = 0.016) and 45° (*P* = 0.033) (Table 5). The control group showed significantly higher length change rates at an ankle joint plantar flexion of 0° than at an ankle joint plantar flexion of 20°. Among women, no significant difference was observed at any of the plantar flexion angles between the control and CAI groups.

**Table 5:** Average dehiscence rate between the fibular malleolus and talus by ankle joint angle during stress

Joint angle		Men control (mm)	Men CAI (mm)	*P* value	Effect size
Anterior	0°	6.81 ± 3.92	7.50 ± 4.44	*P* = 0.102	0.24
	20°	3.26 ± 2.18	7.41 ± 3.99	*P = 0.016	0.09
	45°	3.95 ± 2.11	6.77 ± 3.49	*P = 0.033	0.03
Inversion	0°	5.53 ± 2.53	7.45 ± 4.51	*P* = 0.086	0.32
	20°	3.44 ± 1.95	5.70 ± 2.16	*P = 0.029	0.15
	45°	4.19 ± 2.26	4.98 ± 2.51	*P* = 1.005	0.22
Joint angle		Women control (mm)	Women CAI (mm)	*P* value	Effect size
Anterior	0°	5.46 ± 2.10	4.97 ± 0.95	*P* = 0.098	0.06
	20°	5.65 ± 4.18	4.54 ± 2.84	P = 0.102	0.11
	45°	6.20 ± 4.55	5.24 ± 2.66	*P* = 0.987	0.22
Inversion	0°	7.83 ± 4.66	5.32 ± 2.39	*P* = 0.808	0.31
	20°	8.31 ± 5.38	5.23 ± 2.62	*P* = 0.955	0.26
	45°	5.73 ± 2.98	5.67 ± 2.65	*P* = 1.009	0.28

*CAI* chronic ankle instability

*Significant difference between groups (P < 0.05)

### Length change rates of the fibular lateral malleolus and talus during the inversion stress test

#### Data according to sex

Among men, length change rates between the fibular lateral malleolus and talus during the inversion stress test were significantly higher in the CAI group at an ankle joint plantar flexion of 20° (*P* = 0.029) (Table 5). The control group showed significantly higher length change rates at an ankle joint plantar flexion of 0° than at an ankle joint plantar flexion of 20°. Among women, no significant difference was observed at any of the plantar flexion angles between the control and CAI groups.

## Discussion

Our findings suggest that the anterior drawer and inversion stress tests are effective for determining length change rates between the fibular lateral malleolus and talus at an ankle joint plantar flexion of 20° in men. This may be the best ankle joint angle for the measurement of length change rates. Lee et al. evaluated the ATFL after ankle sprains and examined changes in the distance between the fibular lateral malleolus and talus during stress tests using diagnostic ultrasound [17]. Sisson et al. also conducted quantitative evaluations of the ankle joint using Telos and ultrasound [23]. In the present study, we compared the data of a control group comprising subjects with no history of ankle sprains or lower limb surgery to the data of a CAI group comprising subjects with ankle joint instability. We were able to clarify the differences in length change rates between the fibular lateral malleolus and talus using anterior drawer and inversion stress tests and the differences in ankle joint plantar flexion angles using Telos and diagnostic ultrasound.

The distance between the fibular lateral malleolus and talus at rest was significantly higher in the CAI group in both men and women. A previous study by Croy et al. indicated no significant difference in the distance between the fibular lateral malleolus and talus at rest (18.6 ± 1.5 mm in the control group and 18.8 ± 2.1 mm in the CAI group) [18]. This result was different from that of our study. The ATFL adheres to the fibular lateral malleolus and talus and prevents dehiscence [9]. As the ATFL becomes flaccid with repetitive ankle sprains, there is a high possibility that this might have been the reason for the greater distance between the fibular lateral malleolus and talus in the CAI group. In addition, the distance between these structures at rest was significantly higher among men in the control and CAI groups than among women in both groups. With changes in distance, the influence of the shapes of the fibular lateral malleolus and talus may come into play. Taser et al. reported that the width of the fibular lateral malleolus at 1 cm distal to the tibial plafond was significantly greater in men than in women [24]. Lee et al. reported that the shape of the fibular lateral malleolus and talus is different among individuals [17]. It is possible that there are sex-related differences in bone shapes among men and women, which is the reason why we strongly feel that different evaluation methods are required for men and women.

Differences in length change rates between the fibular lateral malleolus and talus during the anterior drawer stress test were not significantly different between the CAI and control groups at an ankle joint plantar flexion of 0°. Croy et al. reported that the anterior dehiscence rates of the fibular lateral malleolus and talus were significantly higher in the CAI group than in the control group when anterior drawer stress test was performed at an ankle joint plantar flexion of 0° [18]. Anatomically, the ATFL, which is adherent to the fibular lateral malleolus and talus, tenses during ankle plantar flexion and relaxes during dorsiflexion [9]. Furthermore, a cadaver study showed that during ATFL dissection, the trochlea of the talus moved inward with the inner deltoid ligament as the axis while rotating inward [24]. This shows that the ATFL is flaccid in the intermediate ankle joint position, which may be why dehiscence of the fibular lateral malleolus and talus increased in the control group and no significant difference was observed between the CAI and control groups. However, the length change rates of the fibular lateral malleolus and talus were significantly higher in the CAI group than in the control group at ankle joint plantar flexions of 20° and 45° in men. The ATFL functionally slows or stops the forward movement of the trochlea of the talus in the plantar flexion position [25] and limits the dehiscence of the fibular lateral malleolus and talus in ankle plantar flexion. However, in the CAI group, the distance restricting the dehiscence of these structures increased because of the flaccid nature of the ATFL. Therefore, we considered this to be the reason for the decrease in the fibular lateral malleolus and talus dehiscence rates at plantar flexions of 20° and 45° compared with those at 0° in the anterior drawer stress test.

Conversely, length change rates of the fibular lateral malleolus and talus during the inversion stress test were lower among men in the control group than in the CAI group at an ankle joint plantar flexion of 20°. Under inversion stress, the ATFL becomes tenser with ankle joint plantar flexion and inversion. The calcaneofibular ligament (CFL) also becomes tenser with inversion [9]. The CFL originates from the anterior end of the fibula below the ATFL attachment and runs obliquely backward, approximately 130° with respect to the long axis of the fibula. Thus, it becomes tense with dorsiflexion and inversion [9]. It is thought that when the ATFL and CFL are tense, they restrict the length change rates during ankle joint plantar flexion and inversion. Therefore, men in the control group had significantly lower length change rates between the fibular lateral malleolus and talus at an ankle joint plantar flexion of 20°. In addition, Croy et al. reported that inversion stress at an ankle joint plantar flexion of 30° in their CAI group was associated with significantly high length change rates [8]. Based on this information, we considered that the rate of inversional dehiscence between the fibular lateral malleolus and talus increased in the CAI group because of the flaccidity of the ATFL under inversion stress and the absence of movement cessation of the talus.

In this study, no significant difference in anterior drawer and inversion stress test results was observed between women in the control group and those in the CAI group. Ventura et al. reported no significant differences among women in their sprain and non-sprain groups with respect to the rate of positivity of the anterior drawer test for the talus on stress radiography [26]. That study and ours showed similar results, suggesting that both the ATFL and movement cessation caused by the CFL contribute to the function of the fibular lateral malleolus and talus. The effects caused by these ligaments are stronger in women than in men.

This study had several limitations. First, we did not evaluate the function of the ATFL itself. Second, length change rates were evaluated using the fibular lateral malleolus and talus as bone markers. It is possible that the ATFL may not have been damaged in our subjects and that the length change rate between the fibular lateral malleolus and talus increased because of joint laxity. It is difficult to judge whether the effects of joint laxity resulted in CAI. This issue should be addressed in future studies.

## Conclusions

In this study, men in the CAI group showed significantly higher length change rates of the fibular lateral malleolus and talus than those in the control group at an ankle joint plantar flexion of 20° in the anterior drawer and inversion stress tests. This suggests that an ankle joint plantar flexion of 20° may be the best angle for the performance of anterior drawer and inversion stress tests in men and should be considered for use in CAI evaluations. No significant difference in length change rates based on the ankle plantar flexion position was observed among women between the control and CAI groups. Further examinations that take joint laxity into account are required in the future.

## Abbreviations

ATFL: anterior talofibular ligament
CAI: chronic ankle instability
CFL: calcaneofibular ligament
ICC: intraclass correlation coefficients
MRI: magnetic resonance imaging
